# Supplementary material for: Prioritizing autoimmunity risk variants for functional analyses by fine-mapping mutations under natural selection
Source: Nat Commun. 2022 Nov 18;13:7069. doi: 10.1038/s41467-022-34461-9 (PMC9674589; doi:10.1038/s41467-022-34461-9)
Supplement: Supplementary file 1 — Supplementary Information [file 41467_2022_34461_MOESM1_ESM.pdf]

Prioritising Autoimmunity Risk Variants for Functional Analyses by Fine-Mapping Mutations Under Natural Selection

Supplementary Information

Supplementary Figures

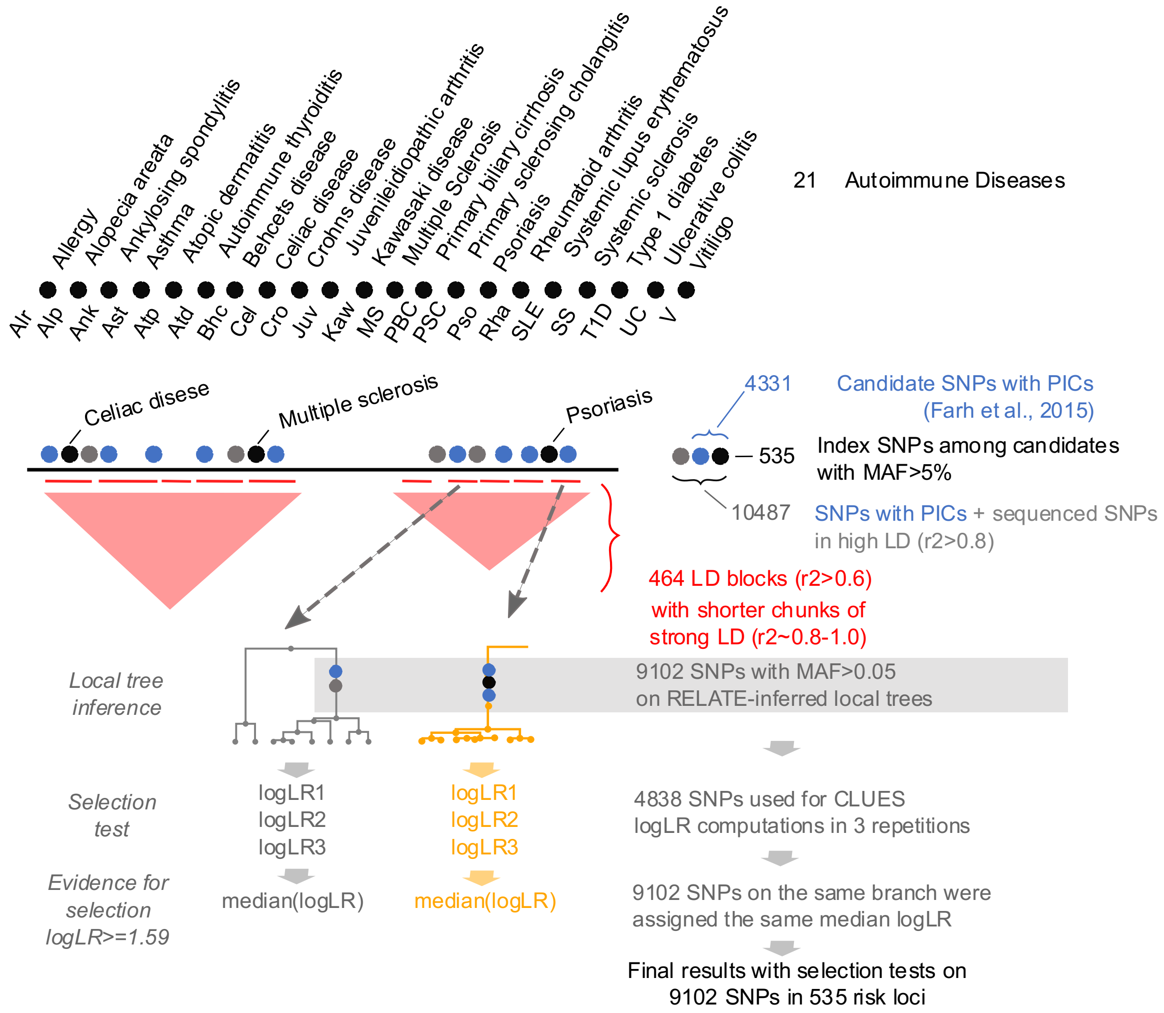

Supplementary Figure 1 Schematic overview of the study workflow

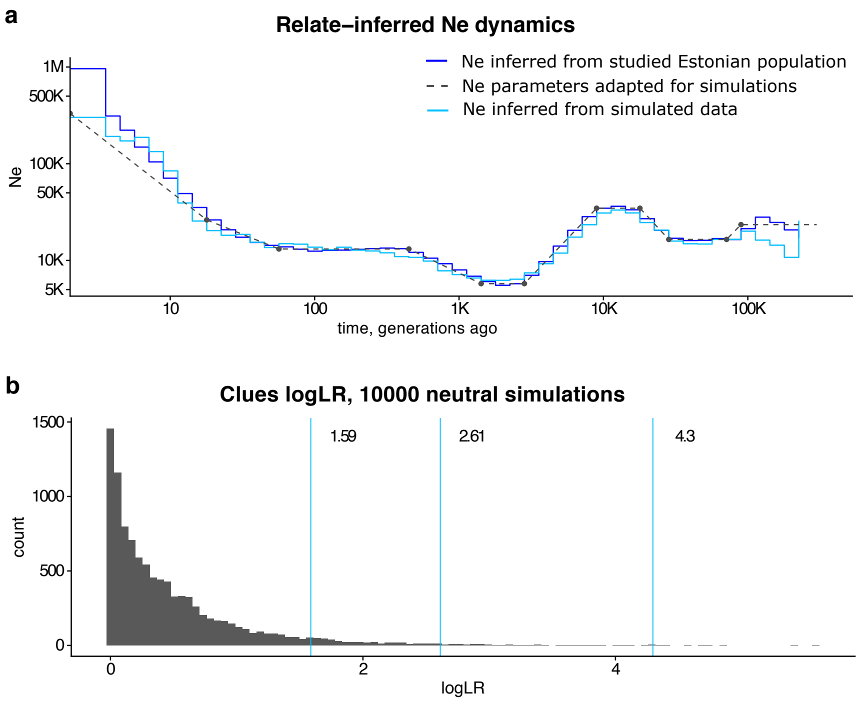

Supplementary Figure 2 Relate–inferred history of effective population size (Ne) for Estonian population and logLR distribution based on simulations

a Effective population size (Ne) is plotted against time in human generations.

Ne values abbreviated as 5K and 1M should be read as 5 thousand (kilo) and one million (mega) individuals, respectively. Line in dark blue shows Ne trajectory inferred from the Estonian population using *Relate*. The dashed line in grey shows Ne parameters adapted and used for simulations. Line in light blue shows Ne trajectory inferred from simulated data using *Relate*. **b** Distribution of logLR from 10000 simulated datasets. The three vertical lines in light blue show 95%, 99%, and 99.9% percentile points, respectively. We use the 95% percentile point of 1.59 as our neutrality rejection threshold. Raw data are provided in Supplementary Data 8.

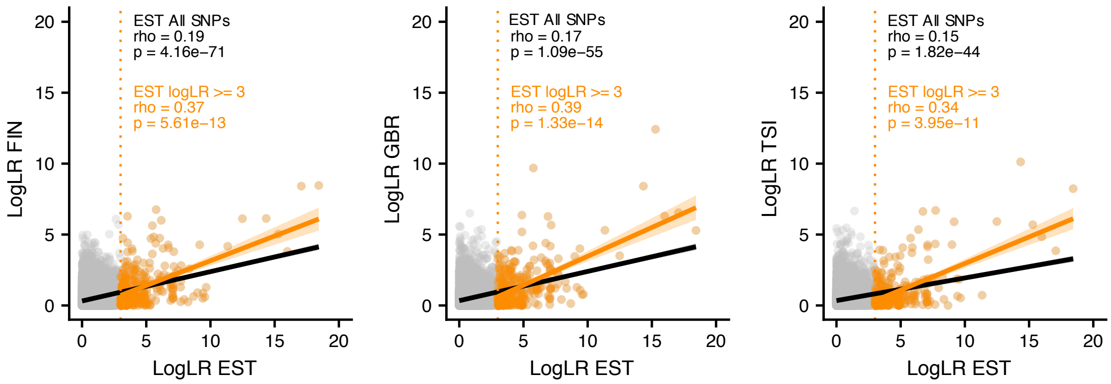

Supplementary Figure 3 Reproducibility of selection signals (logLR ≥ 3) between Estonians and European populations. SNP-wise logLR estimates in Estonians are compared to that in Finns (FIN), British (GBR) and Italians (TSI). Spearman correlation (rho and p-value in orange) is computed separately for SNPs with strong evidence for selection in Estonians (points in orange, logLR ≥ 3) and for all the SNPs (rho and p-value in black). Nominal p-values are reported with no multiple testing correction. The trend line is obtained by fitting a generalized additive model to the corresponding data and the shaded area corresponds to its' 95% confidence interval. Raw data are provided in Supplementary Data 2.

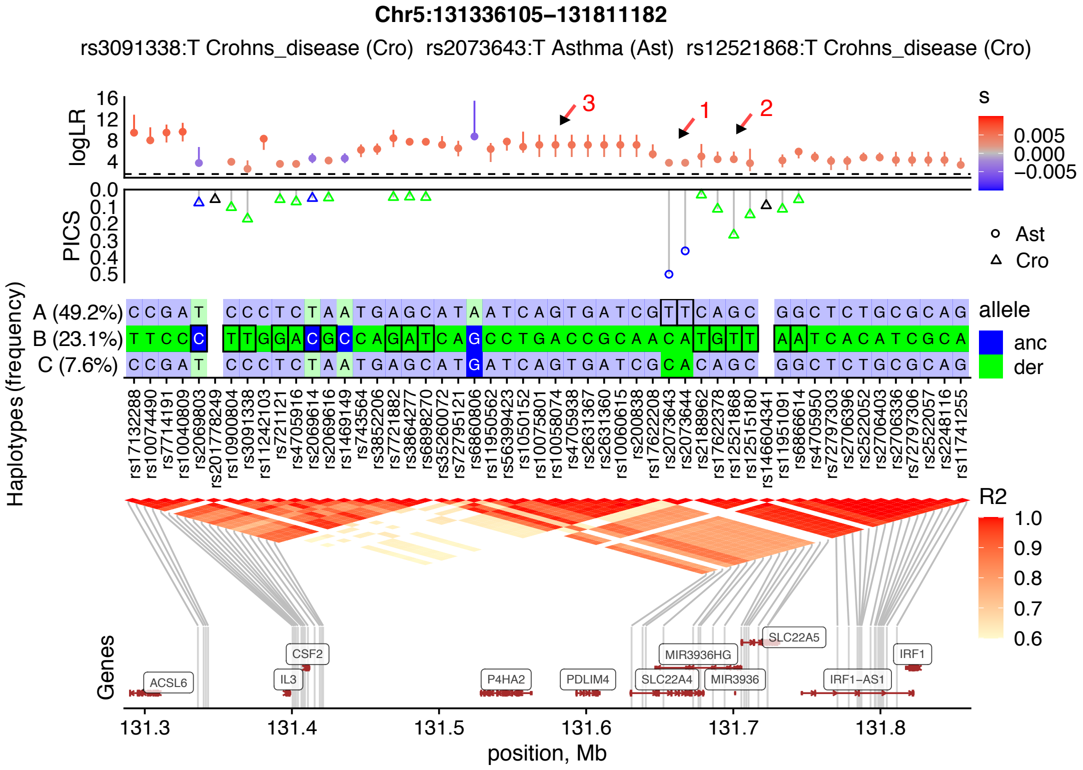

Supplementary Figure 4 LD block with multiple selection signals

Top candidate SNPs for asthma and Crohn's disease (First and second red arrows), when considered locally, show evidence for selection, but nearby SNPs (Third arrow) in linkage disequilibrium ($r2>0.6$) show stronger evidence for selection.

Graph rows represent five layers of annotation, from top to down - logLR, PICS, Haplotypes, LD heatmap, and Genes. The top row shows median logLR estimates with whiskers indicating minimum and maximum of three estimates. Whisker colour reflects the sign and magnitude of the selection coefficient estimated on the SNP derived allele. The dashed line separates LogLR values (below) expected under the neutral demographic history of Estonians. The values above suggest evidence for selection. The second row shows PICS scores for each candidate SNP reported in Farh et al.[1]. Candidate SNPs for multiple diseases are shown with different symbols. The symbol is blue when the risk allele is ancestral and green when derived. The next row shows the haplotype structure in the LD-block with respect to ancestral (blue) and derived alleles (green). Only the most common haplotypes (A, B and C) are shown with frequencies (in brackets) estimated in 1800 Estonians. SNPs with $\text{logLR} \geq 1.59$ are highlighted with darker colours, and risk alleles are indicated with solid margins. The fourth row shows a heatmap of pairwise linkage disequilibrium between all the candidate SNPs from Farh et al.[1] even if they have $r2 < 0.6$, which defines an LD-block region. The bottom row depicts gene annotations from the Ensembl genome database (version 87, Human genome build GRCh37). Raw data are provided in Supplementary Data 2.

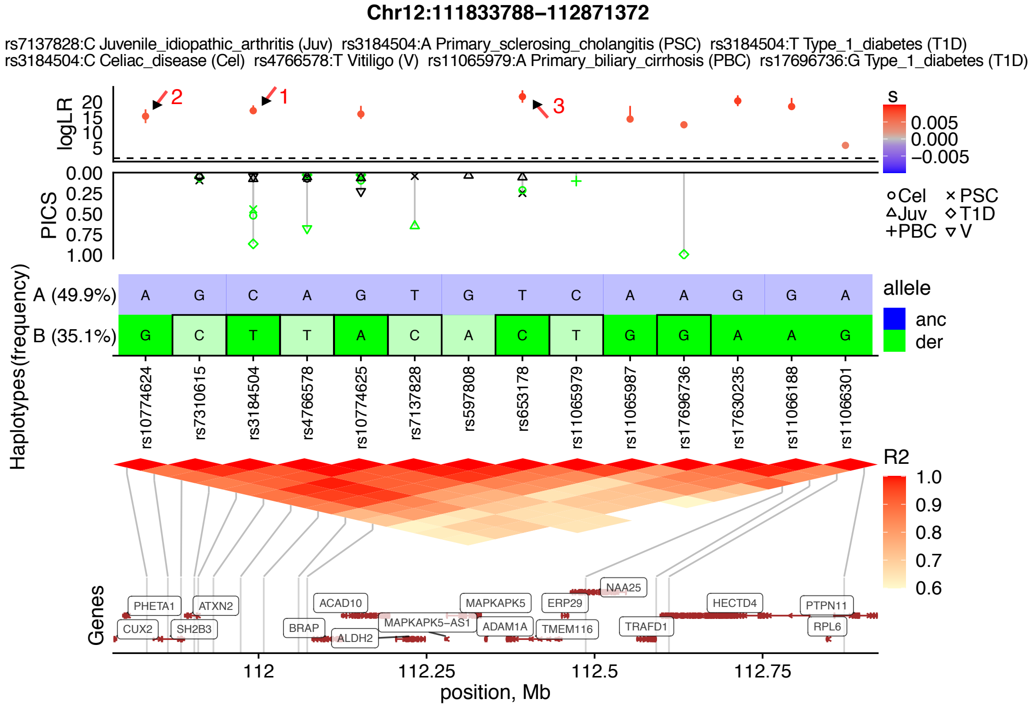

Supplementary Figure 5 Local tree-based analysis distinguishes novel selection targets in previously reported selection signals.

Previously reported selection target (rs3184504) in celiac disease risk loci (First red arrow) is in strong LD with two novel SNPs, candidates for selection targets (Second and third red arrows). Graph rows represent five layers of annotation, from top to down - logLR, PICS, Haplotypes, LD heatmap, and Genes. The top row shows median logLR estimates with whiskers indicating minimum and maximum of three estimates. Whisker colour reflects the sign and magnitude of the selection coefficient estimated on the SNP derived allele. The dashed line separates LogLR values (below) expected under the neutral demographic history of Estonians. The values above suggest evidence for selection. The second row shows PICS values for each candidate SNP reported in Farh et al.[1]. Candidate SNPs for multiple diseases are shown with different symbols. The symbol is blue when the risk allele is ancestral and green when derived. The next row shows the haplotype structure in the LD-block with respect to ancestral (blue) and derived alleles (green). Only the most common haplotypes (A, B and C) are shown with frequencies (in brackets) estimated in 1800 Estonians. SNPs with logLR ≥ 1.59 are highlighted with darker colours, and risk alleles are indicated with solid margins. The fourth row shows a heatmap of pairwise linkage disequilibrium between all the candidate SNPs from Farh et al.[1] even if they have r2 < 0.6, which defines an LD-block region. The bottom row depicts gene annotations from the Ensembl genome database (version 87, Human genome build GRCh37). Raw data are provided in Supplementary Data 2.

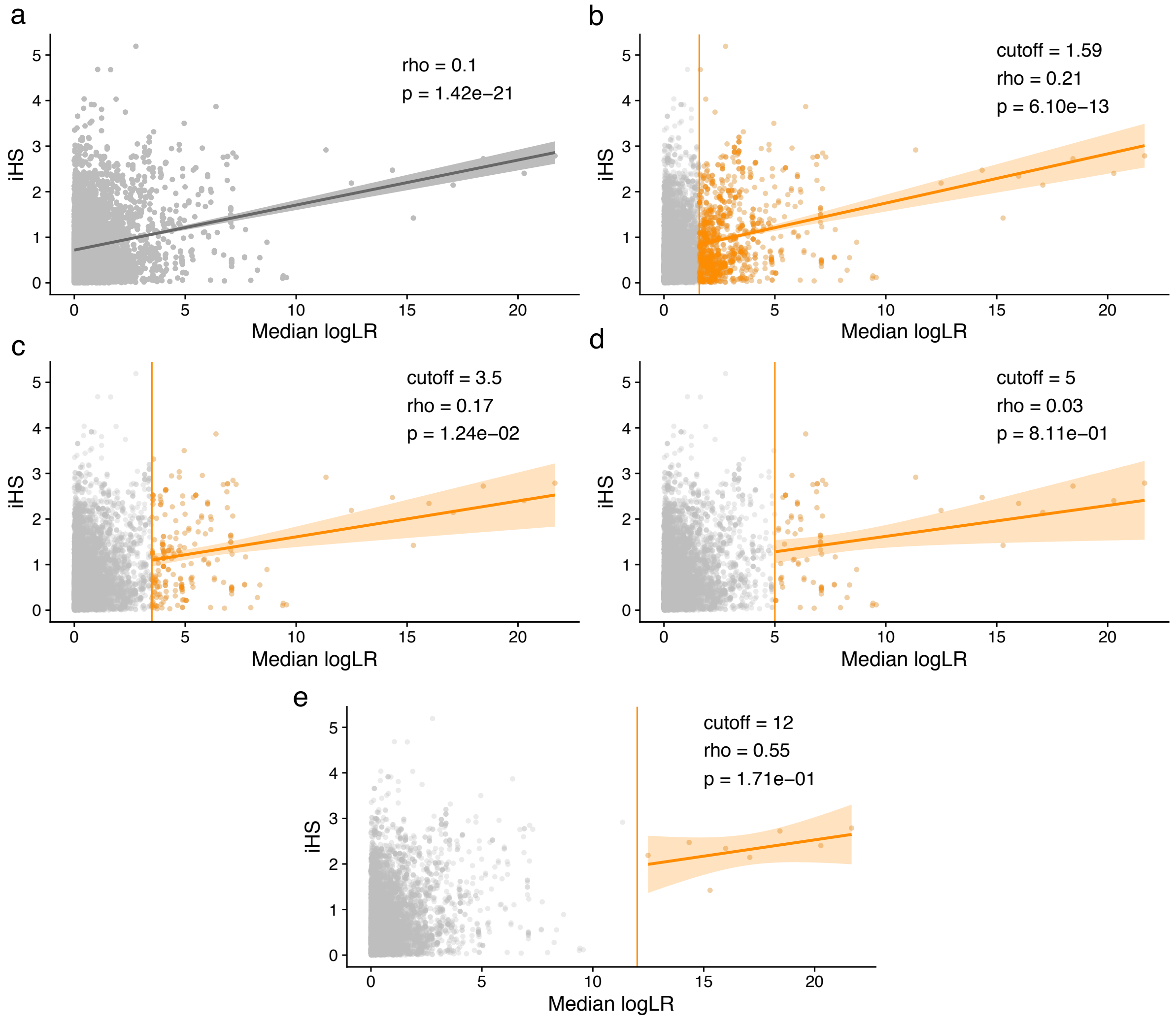

Supplementary Figure 6 Correlation between haplotype-based iHS-score and local-tree based logLR.

Spearman correlation (rho) between iHS-score and logLR when considering all the analyzed SNPs (panel a) and when considering subsets of SNPs with increasing strength of selection (panels **b, c, d,** and **e** at increasing logLR cutoffs). Nominal p-values are reported with no multiple testing correction. The trend line is obtained by fitting a generalized additive model to the corresponding data and the shaded area corresponds to its' 95% confidence interval. Raw data are provided in Supplementary Data 2.

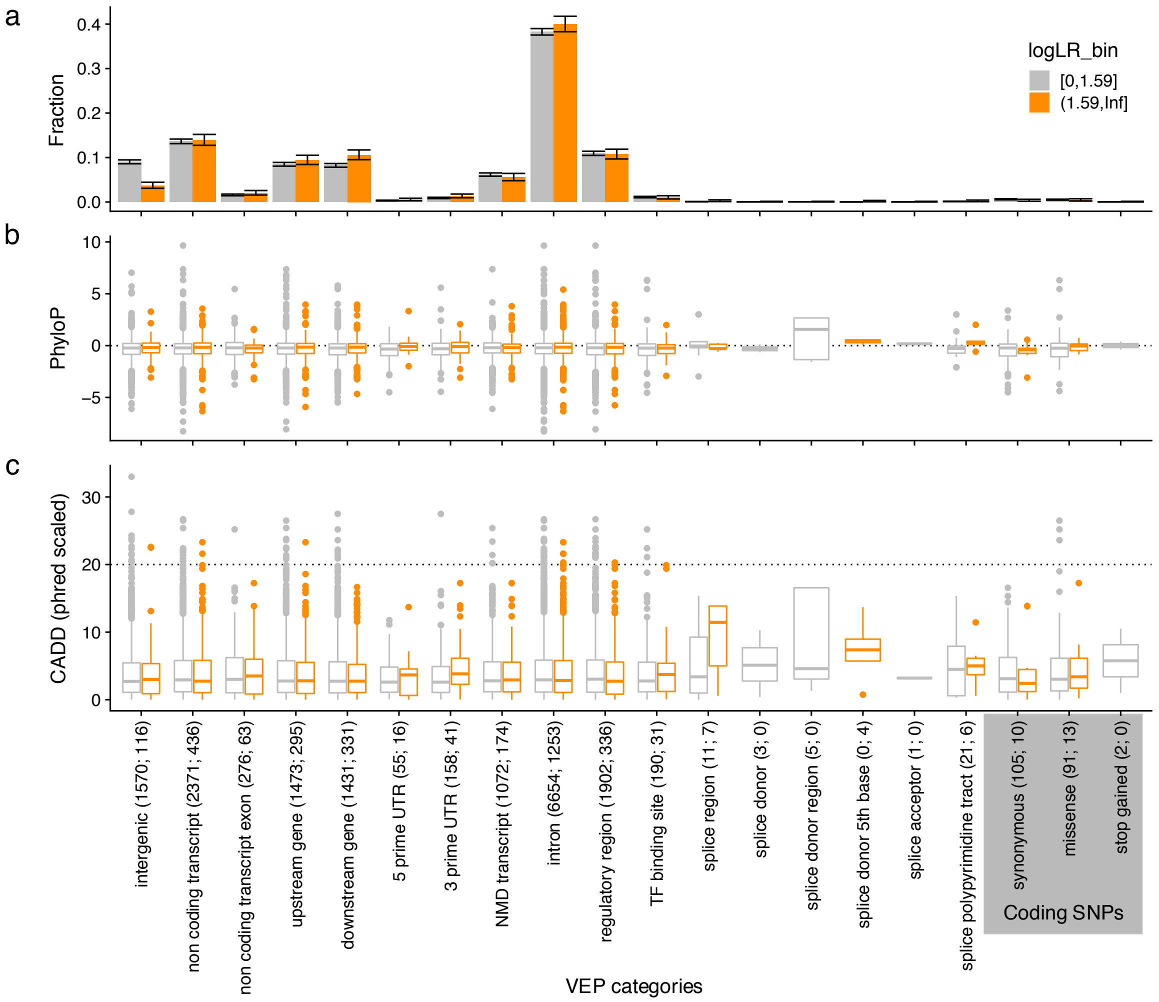

Supplementary Figure 7 Predicted deleteriousness, evolutionary conservation and SNP effect at gene, transcript and protein levels.

a Distribution of candidate SNPs among VEP functional categories (version 106)[2]. Grey bars show the fraction of SNPs with logLR < 1.59 (i.e., with no evidence for selection) falling into each category listed on the x axis while orange bars show the same distribution for SNPs with logLR ≥ 1.59 (i.e., potential selection targets). Whiskers represent 95% confidence interval. The numbers in brackets on the x axis show the number of SNPs (n) in each category with the first number corresponding to the grey and the second – to the orange bar. If a SNPs fell into more than one VEP category such annotations were counted individually and hence the total sums used to calculate the fraction were 17391 and 3132 for SNP with logLR < 1.59 and logLR

≥ 1.59 accordingly. **b** PhyloP evolutionary conservation scores for candidate SNPs. Positive scores suggest conservation (slower than expected under neutral drift), and negative values indicate acceleration (faster than expected). **c** Phred-scaled CADD scores for SNPs predicting deleteriousness. A dotted horizontal line separates strongly deleterious SNPs. In both **b** and **c** boxes show the 25th, 50th and 75th quantiles; whiskers show values within 1.5 times the interquartile range (IQR), and individual dots are values outside this range.
Raw data are provided in Supplementary Data 2.

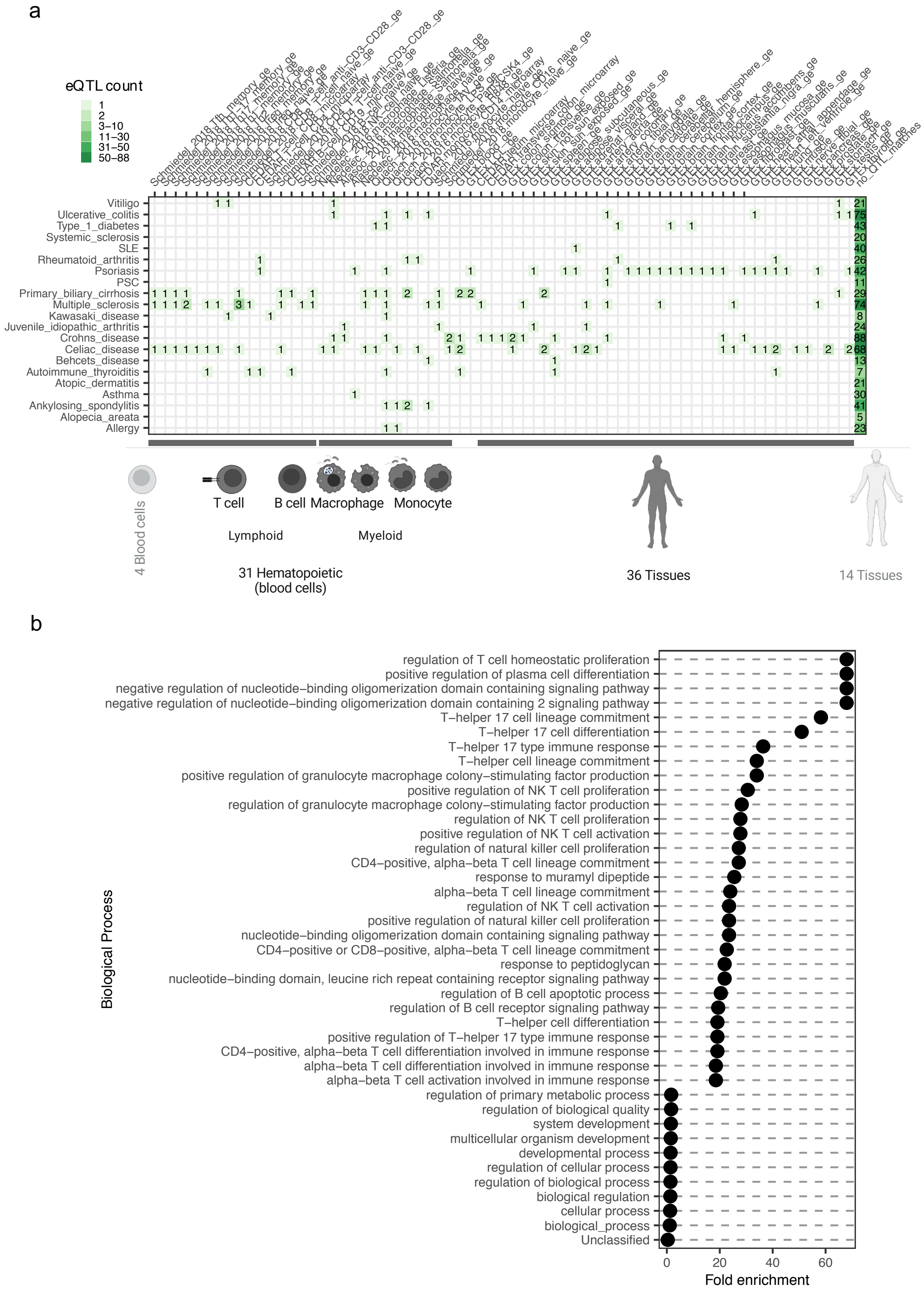

Supplementary Figure 8 Diseases and number of associated candidate SNPs matching eQTLs.

a Heatmap shows the number of candidate SNPs with eQTL for each disease (disease eQTLs) and tissue/cell where eQTL was reported. The last column shows the number of candidate SNPs without eQTL **b** Biological pathways enriched for genes affected by disease eQTLs. Raw data for the figure panel **a** are provided in Supplementary Data 6 and Supplementary Data 7, and for panel b in Source Data file. Created with BioRender.com.

Supplementary Methods

Freely available software and databases used.
Listed in the order they appear in Materials and Methods sections.

Genetic risk loci associated with autoimmune diseases and LD blocks

Haplostrips[3] version 1.2.1
https://bitbucket.org/dmarnetto/haplostrips/src/master/

ieugwasr[4] R package version 0.1.5
https://github.com/MRCIEU/ieugwasr

Handling VCF files, filtering, subsetting datasets and LD calculations

bcftools[5] v. 1.9
https://github.com/samtools/bcftools

vcftools[6] v. 0.1.14
https://vcftools.github.io/downloads.html

Evidence for natural selection based on local trees

Relate[7] version 1.1.4
https://myersgroup.github.io/relate/
CLUES[8] version 1.0
https://github.com/standard-aaron/clues

Preparing datasets and running the Relate/CLUES analyses

Conversion from vcf to haps/sample

```
for chr in {1..22}
do
        path-to-relate/bin/RelateFileFormats \
                --mode ConvertFromVcf \
                --haps ${haplotypes}_${chr}.haps \
                --sample ${haplotypes}_${chr}.sample \
                -i input_${vcf}_${chr} \
                --chr $chr
done
```

Preparing the input files for Relate

```
for chr in {1..22}
do
```

```
        path-to-relate/scripts/PrepareInputFiles/PrepareInputFiles.sh \
                --haps ${haplotypes}_${chr}.haps \
                --sample ${haplotypes}_${chr}.sample \
                --ancestor homo_sapiens_ancestor_${chr}.fa \
                --mask strict_mask.chr${chr}.fasta.gz \
                --poplabels Estonians_2305.poplabels \
                -o ${haplotypes}_prep_chr${chr}
done
```

# Tree building

```
path-to-relate/scripts/RelateParallel/RelateParallel.sh \
        -m 1.25e-8 \
        -N 30000 \
        --haps  ${haplotypes}_prep_chr${chr}.haps.gz \
        --sample  ${haplotypes}_prep_chr${chr}.sample.gz \
        --annot  ${haplotypes}_prep_chr${chr}.annot \
        --dist  ${haplotypes}_prep_chr${chr}.dist.gz \
        --map genetic_map_chr${chr}.txt.gz \
        --threads 10 \
        -o Relate_trees_Estonians_2305_chr${chr}
```

# Extracting sustrees for sample subsets

```
for pop in {100,1800}
do
for chr in {1..22}
do
        path-to-relate/bin/RelateExtract \
                --mode SubTreesForSubpopulation \
                --anc Relate_trees_Estonians_2305_chr${chr}.anc.gz \
                --mut Relate_trees_Estonians_2305_chr${chr}.mut.gz \
                --poplabels Estonians_2305.poplabels \
                --pop_of_interest Estonians_${pop} \
                -o Relate_trees_Estonians_${pop}_chr${chr}
done
done
```

# Estimating population size trajectory

```
path-to-relate/scripts/EstimatePopulationSize/EstimatePopulationSize.sh \
        -i Relate_trees_Estonians_100 \
        --first_chr 1
        --last_chr 22
        -m 1.25e-8 \
        --poplabels Relate_trees_Estonians_100.poplabels \
        --threads 10 \
        --bins 3,7,0.2 \
        --years_per_gen 28 \
        --num_iter 5 \
        --threshold 0.5 \
        -o Relate_trees_Estonians_100_popsize
```

# Sample branch length for CLUES for a given position of interest defined with $chr and $pos

```
path-to-relate/scripts/SampleBranchLengths/SampleBranchLengths.sh \
        -i Relate_trees_Estonians_1800_chr${chr} \
        -o tree_chr${chr}_pos${pos}_200_samples \
        -m 1.25e-8 \
        --coal Relate_trees_Estonians_100_popsize.coal \
        --format b \
        --num_samples 200 \
        --first_bp ${pos} \
        --last_bp ${pos}
```

# Run CLUES for a given position of interest defined with $chr and $pos

```
python3 programs/clues-master/inference.py \
        --times tree_chr${chr}_pos${pos}_200_samples \
        --coal Relate_trees_Estonians_100_popsize.coal  \
        --thin 10 \
        --burnin 100 \
        --tCutoff 1000 \
        --out chr${chr}_pos${pos}_samples200 >
        chr${chr}_pos${pos}_log.txt
```

Simulating population history

msprime[9] version 0.7.4
https://tskit.dev/msprime/docs/stable/installation.html

Positive selection test based on iHS score

Selscan[10] program version 1.2.1a
https://github.com/szpiech/selscan

Inferring eQTLs, target genes and tissues using eQTL Catalogue

qvalue[11] R package version 2.28.0
http://github.com/jdstorey/qvalue

PANTHER[12] Overrepresentation Test
http://pantherdb.org/tools/compareToRefList.jsp

Basic statistical tests and graphs

stats package in R[13] version 4.2.0
https://www.R-project.org/

ggplot2[14] version 3.3.6
https://ggplot2.tidyverse.org/

Databases used:

The GO Biological Process annotation dataset
https://zenodo.org/record/6399963

1000G strict mask
http://ftp.1000genomes.ebi.ac.uk/vol1/ftp/release/20130502/supporting/accessible_genome_masks/StrictMask/

Ancestral Genome
http://ftp.1000genomes.ebi.ac.uk/vol1/ftp/phase1/analysis_results/supporting/ancestral_alignments/

Recombination map
http://ftp.1000genomes.ebi.ac.uk/vol1/ftp/technical/working/20110106_recombination_hotspots/

CADD[15] version 1.6
https://cadd.gs.washington.edu/download

PhyloP[16]
http://hgdownload.soe.ucsc.edu/goldenPath/hg19/phyloP100way/

OpenGWAS
https://gwas.mrcieu.ac.uk/

eQTL Catalogue[17]
https://www.ebi.ac.uk/eqtl/
(See Supplementary Data 6 for cell/tissue-specific datasets used in this study)

VEP[2] version 106
https://www.ensembl.org/info/docs/tools/vep/index.html

References

1. Farh, K. K.-H. *et al.* Genetic and epigenetic fine mapping of causal autoimmune disease variants. *Nature* **518**, 337–343 (2015).
2. McLaren, W. *et al.* The Ensembl Variant Effect Predictor. *Genome Biol.* **17**, 122 (2016).
3. Marnetto, D. & Huerta-Sánchez, E. Haplostrips : revealing population structure through haplotype visualization. *Methods Ecol. Evol.* **8**, 1389–1392 (2017).
4. Elsworth, B. *et al.* The MRC IEU OpenGWAS data infrastructure. *bioRxiv* 2020.08.10.244293 (2020) doi:10.1101/2020.08.10.244293.
5. Danecek, P. *et al.* Twelve years of SAMtools and BCFtools. *Gigascience* **10**, (2021).
6. Danecek, P. *et al.* The variant call format and VCFtools. *Bioinformatics* **27**, 2156–2158 (2011).
7. Speidel, L., Forest, M., Shi, S. & Myers, S. R. A method for genome-wide genealogy estimation for thousands of samples. *Nat. Genet.* **51**, 1321–1329 (2019).
8. Stern, A. J., Wilton, P. R. & Nielsen, R. An approximate full-likelihood method for inferring selection and allele frequency trajectories from DNA sequence data. *PLoS Genet.* **15**, e1008384 (2019).
9. Kelleher, J., Etheridge, A. M. & McVean, G. Efficient Coalescent Simulation and Genealogical Analysis for Large Sample Sizes. *PLoS Comput. Biol.* **12**, e1004842 (2016).
10. Szpiech, Z. A. & Hernandez, R. D. selscan: an efficient multithreaded program to perform EHH-based scans for positive selection. *Mol. Biol. Evol.* **31**, 2824–2827 (2014).

11. Storey, J. D., Bass, A. J., Dabney, A. & Robinson, D. qvalue: Q-value estimation for false discovery rate control. Preprint at http://github.com/jdstorey/qvalue (2022).

12. Thomas, P. D. *et al.* PANTHER: Making genome-scale phylogenetics accessible to all. *Protein Sci.* **31**, 8–22 (2022).

13. R Core Team. R: A Language and Environment for Statistical Computing. Preprint at https://www.R-project.org/ (2022).

14. Wickham, H. ggplot2: Elegant Graphics for Data Analysis. Preprint at https://ggplot2.tidyverse.org (2016).

15. Kircher, M. *et al.* A general framework for estimating the relative pathogenicity of human genetic variants. *Nat. Genet.* **46**, 310–315 (2014).

16. Pollard, K. S., Hubisz, M. J., Rosenbloom, K. R. & Siepel, A. Detection of nonneutral substitution rates on mammalian phylogenies. *Genome Res.* **20**, 110–121 (2010).

17. Kerimov, N. *et al.* A compendium of uniformly processed human gene expression and splicing quantitative trait loci. *Nat. Genet.* **53**, 1290–1299 (2021).
