# Supplementary material for: Prioritizing autoimmunity risk variants for functional analyses by fine-mapping mutations under natural selection
Source: Nat Commun. 2022 Nov 18;13:7069. doi: 10.1038/s41467-022-34461-9 (PMC9674589; doi:10.1038/s41467-022-34461-9)
Supplement: Supplementary file 3 — Description of Additional Supplementary Files [file 41467_2022_34461_MOESM3_ESM.pdf]

Description of Additional Supplementary Files

File Name: Supplementary Data 1
Description: **Total set of 10487 candidate SNPs for 21 inflammatory diseases.**

File Name: Supplementary Data 2
Description: **CLUES-based selection tests for 9102 candidate SNPs mapped on RELATE trees with VEP annotations.** Note that 9102 unique SNPs have 9235 entries (rows) in this table. This is because some SNPs can be associated with multiple diseases. As a result, such SNPs would have distinct PICS scores for different diseases and multiple entries.

File Name: Supplementary Data 3
Description: **Evolutionary scenarios.** Classifications at LD block level and disease loci accompany Figure 3

File Name: Supplementary Data 4
Description: **Revisiting published evidence on selection in inflammatory disease risk loci.**
[a]CLUES-based selection tests from this study. Evidence for selection, logLR ≥ 1.59
[b]Selection test based on iHS from Raj et al 2013. Evidence for selection, |iHS ≥ 2|

File Name: Supplementary Data 5
Description: **Candidate SNPs in balancing haplotypes.** Data on balancing haplotypes from (Siewert & Voight, 2017).
[a]Start and End positions are taken from the 'SNPs_in_Haplotype' field of 'TopScoringHapsAllChromIncXCEU.txt' file.
[b,c]Inferences on Age and logLR are taken from CLUES analysis. We note that such inferences are not applicable for balancing haplotypes and are given for general interest.

File Name: Supplementary Data 6
Description: **Expression QTL datasets searched for candidate SNP matches.** eQTL summary data for each tissue/cell type was retrieved from the eQTL Catalogue project (https://www.ebi.ac.uk/eqtl/). eQTL Catalogue stores uniformly processed gene expression and splicing QTLs from published studies.

File Name: Supplementary Data 7
Description: **Candidate SNPs annotated with matching eQTLs.** There are only 919 matching eQTLs significant at FDR 10%

File Name: Supplementary Data 8
Description: **Effective population size (Ne) parameters inferred for Estonian population**
