# Supplementary material for: Prioritizing autoimmunity risk variants for functional analyses by fine-mapping mutations under natural selection
Source: Nat Commun. 2022 Nov 18;13:7069. doi: 10.1038/s41467-022-34461-9 (PMC9674589; doi:10.1038/s41467-022-34461-9)
Supplement: Supplementary file 12 — Reporting Summary [file 41467_2022_34461_MOESM12_ESM.pdf]

nature portfolio

Corresponding author(s): Bayazit Yunusbayev

Last updated by author(s): Oct 14, 2022

Reporting Summary

Nature Portfolio wishes to improve the reproducibility of the work that we publish. This form provides structure for consistency and transparency in reporting. For further information on Nature Portfolio policies, see our Editorial Policies and the Editorial Policy Checklist.

Statistics

For all statistical analyses, confirm that the following items are present in the figure legend, table legend, main text, or Methods section.

n/a	Confirmed	
☐	☒	The exact sample size (*n*) for each experimental group/condition, given as a discrete number and unit of measurement
☒	☐	A statement on whether measurements were taken from distinct samples or whether the same sample was measured repeatedly
☐	☒	The statistical test(s) used AND whether they are one- or two-sided<br>*Only common tests should be described solely by name; describe more complex techniques in the Methods section.*
☒	☐	A description of all covariates tested
☐	☒	A description of any assumptions or corrections, such as tests of normality and adjustment for multiple comparisons
☐	☒	A full description of the statistical parameters including central tendency (e.g. means) or other basic estimates (e.g. regression coefficient) AND variation (e.g. standard deviation) or associated estimates of uncertainty (e.g. confidence intervals)
☐	☒	For null hypothesis testing, the test statistic (e.g. *F*, *t*, *r*) with confidence intervals, effect sizes, degrees of freedom and *P* value noted<br>*Give P values as exact values whenever suitable.*
☐	☒	For Bayesian analysis, information on the choice of priors and Markov chain Monte Carlo settings
☒	☐	For hierarchical and complex designs, identification of the appropriate level for tests and full reporting of outcomes
☒	☐	Estimates of effect sizes (e.g. Cohen's *d*, Pearson's *r*), indicating how they were calculated

Our web collection on statistics for biologists contains articles on many of the points above.

Software and code

Policy information about availability of computer code

Data collection	No software was used for data collection
Data analysis	The following software was used for data analysis:<br>Handling VCF files, filtering and subsetting and LD calculations:<br>bcftools v. 1.9 (https://github.com/samtools/bcftools)<br>vcftools v. 0.1.14 (https://vcftools.github.io/downloads.html)<br><br>Estimating haplotypes, retrieving risk alleles for risk SNPs:<br>Haplostrips version 1.2.1 (https://bitbucket.org/dmarnetto/haplostrips/src/master/)<br>ieugwasr R package version 0.1.5 (https://github.com/MRCIEU/ieugwasr)<br><br>Natural selection analysis:<br>Relate v. 1.1.4 (https://myersgroup.github.io/relate/)<br>CLUES v 1.0 (https://github.com/35ajstern/clues)<br>Selscan program version 1.2.1a (https://github.com/szpiech/selscan)<br><br>Estimating false discovery rate:<br>qvalue R package (http://github.com/jdstorey/qvalue)<br><br>Simulating population history:<br>msprime v. 0.7.4 (https://tskit.dev/msprime/docs/stable/installation.html)

Inferring eQTLs, target genes and tissues using eQTL Catalogue:
qvalue11 R package version 2.28.0 (http://github.com/jdstorey/qvalue)
PANTHER12 Overrepresentation Test (http://pantherdb.org/tools/compareToRefList.jsp)

Basic statistical tests and graphs:

stats package in R13 version 4.2.0 (https://www.R-project.org/)
ggplot214 version 3.3.6 (https://ggplot2.tidyverse.org/)

For manuscripts utilizing custom algorithms or software that are central to the research but not yet described in published literature, software must be made available to editors and reviewers. We strongly encourage code deposition in a community repository (e.g. GitHub). See the Nature Portfolio guidelines for submitting code & software for further information.

Data

Policy information about availability of data

All manuscripts must include a data availability statement. This statement should provide the following information, where applicable:

- Accession codes, unique identifiers, or web links for publicly available datasets
- A description of any restrictions on data availability
- For clinical datasets or third party data, please ensure that the statement adheres to our policy

Data availability:

The Estonian Biobank sequencing data analyzed in this study are available upon request. The application procedure to access the data can be found under the following link: https://genomics.ut.ee/en/content/estonian-biobank

Full annotation data for the 153 risk loci with logLR, PICS scores, risk haplotype, Ensembl genes, and LD blocks are available in the Figshare repository, https://figshare.com/s/c6b80455488afbd1655a.

Source data are provided with this paper.

Publicly available databases used:

The GO Biological Process annotation dataset
https://zenodo.org/record/6399963

1000G strict mask
http://ftp.1000genomes.ebi.ac.uk/vol1/ftp/release/20130502/supporting/accessible_genome_masks/StrictMask/

Ancestral Genome
http://ftp.1000genomes.ebi.ac.uk/vol1/ftp/phase1/analysis_results/supporting/ancestral_alignments/

Recombination map
http://ftp.1000genomes.ebi.ac.uk/vol1/ftp/technical/working/20110106_recombination_hotspots/

CADD version 1.6
https://cadd.gs.washington.edu/download

PhyloP
http://hgdownload.soe.ucsc.edu/goldenPath/hg19/phyloP100way/

OpenGWAS
https://gwas.mrcieu.ac.uk/

eQTL Catalogue
https://www.ebi.ac.uk/eqtl/
(See Supplementary Data 6 for cell/tissue-specific datasets used in this study)

VEP version 106
https://www.ensembl.org/info/docs/tools/vep/index.html

Human research participants

Policy information about studies involving human research participants and Sex and Gender in Research.

Reporting on sex and gender

In this study, genetic analyses are based on genomic sequences from autosomal chromosomes and the underlying theory

Reporting on sex and gender	does not require information on the donor's sex. Therefore, no sex- or gender-based analyses were required.
Population characteristics	The Estonian Biobank cohort closely mirrors the entire Estonian population in terms of age, gender and spatial structure. The individuals with whole genome sequences originaly reported in Kals et al., 2019 were selected so as to cover as much of the Estonian diversity as possible, representing both parishes with small population size and big towns.
Recruitment	Estonian Biobank participants are recruited on a fully voluntary basis with no focus on donor's disease. Samples selected for sequencing in Kals et al.2019 were picked based on their ethnicity (Estonians) and place of birth (to ensure small parishes are represented).
Ethics oversight	All Estonian Biobank participants have signed a broad informed consent which allows research in the fields of genetic epidemiology, disease risk factors and population history. All work at Estonian Biobank is conducted according to the Estonian Human Gene Research Act. The original study generating the WGS data (Kals et al., 2019) was approved by the Research Ethics Committee of the University of Tartu (application number 234/T-12).

Note that full information on the approval of the study protocol must also be provided in the manuscript.

Field-specific reporting

Please select the one below that is the best fit for your research. If you are not sure, read the appropriate sections before making your selection.

☐ Life sciences ☐ Behavioural & social sciences ☒ Ecological, evolutionary & environmental sciences

For a reference copy of the document with all sections, see nature.com/documents/nr-reporting-summary-flat.pdf

Ecological, evolutionary & environmental sciences study design

All studies must disclose on these points even when the disclosure is negative.

Study description	Our study is centered around a dataset of whole genome sequences from four populations which we tested for genetic signals of natural selection.
Research sample	In our study we used a previously published dataset of ~2300 whole genome sequences (WGS) of the participants of the Estonian Biobank. We chose this dataset as it contains a relatively high number of WGS coming from a relatively homogenuous population which allows gaining high power when testing for natural selection. For comparison, we also analyzed published dataset on three populations of European ancestry from the 1000 Genomes Project (1000 Genomes Project Consortium, 2015).
Sampling strategy	No sampling was done by us - we are using an already existing dataset. To the best of our knowledge this is one of the biggest WGS datasets used to detect recent natural selection in humans
Data collection	No data collection was done
Timing and spatial scale	No data collection was done
Data exclusions	Individuals were excluded in order to remove relatives as well as genetic outliers based on PCA results and singleton counts. Exclusion criteria were pre-established using prior knowledge on the properties of the dataset (Pankratov et al. 2020). When excluding individuals we were blind to their genotypes at any particular locus.
Reproducibility	We tested reproducibility of selection signals detected in Estonian population in three populations of European ancestry (Finns, British and Italians).
Randomization	In our study, we treat the whole dataset as a single group as we don't do any comparisons or differential treatment. Hence, the idea of randomization is not applicable.
Blinding	In our study we didn't treat any individual sample or sample group separately hence blinding in such sense is not applicable. When filtering the dataset (see data exclusions) we were blind to loci-specific genotypes of the included/excluded samples.

Did the study involve field work? ☐ Yes ☒ No

Reporting for specific materials, systems and methods

We require information from authors about some types of materials, experimental systems and methods used in many studies. Here, indicate whether each material, system or method listed is relevant to your study. If you are not sure if a list item applies to your research, read the appropriate section before selecting a response.

Materials & experimental systems

n/a	Involved in the study
☒	☐ Antibodies
☒	☐ Eukaryotic cell lines
☒	☐ Palaeontology and archaeology
☒	☐ Animals and other organisms
☒	☐ Clinical data
☒	☐ Dual use research of concern

Methods

n/a	Involved in the study
☒	☐ ChIP-seq
☒	☐ Flow cytometry
☒	☐ MRI-based neuroimaging
